# Changes in subset distribution and impaired function of circulating natural killer cells in patients with colorectal cancer

**DOI:** 10.1038/s41598-024-63103-x

**Published:** 2024-05-28

**Authors:** Shujin Zu, Yan Lu, Rui Xing, Xiang Chen, Longyi Zhang

**Affiliations:** 1 Department of Reproductive Center, Affiliated Dongyang Hospital of Wenzhou Medical University, 60 West Wuning Road, Dongyang, 322100 Zhejiang China; 2 Clinical Laboratory, DongYang People’s Hospital, Affiliated Dongyang Hospital of Wenzhou Medical University, 60 West Wuning Road, Dongyang, 322100 Zhejiang China; 3 The Department of Hematology, Affiliated Dongyang Hospital of Wenzhou Medical University, Dongyang, Zhejiang China; 4 Department of Biomedical Sciences Laboratory, Affiliated DongYang Hospital of Wenzhou Medical University, Dongyang, Zhejiang China

**Keywords:** Natural killer cells, Colorectal cancer, Interferon-γ secretion, Granzyme B, Perforin, Tumor stage, Tumour immunology, Colorectal cancer

## Abstract

Natural killer (NK) cells are closely associated with malignant tumor progression and metastasis. However, studies on their relevance in colorectal cancer (CRC) are limited. We aimed to comprehensively analyze the absolute counts, phenotypes, and function of circulating NK cells in patients with CRC using multiparametric flow cytometry. The distribution of NK cell subsets in the peripheral circulation of patients with CRC was significantly altered relative to the control group. This is shown by the decreased frequency and absolute count of CD56^dim^CD16^+^ NK cells with antitumor effects, contrary to the increased frequency of CD56^bright^ NK and CD56^dim^CD16^-^ NK cells with poor or ineffective antitumor effects. NK cells in patients with CRC were functionally impaired, with decreased intracellular interferon (IFN)-γ secretion and a significantly lower percentage of cell surface granzyme B and perforin expression. In addition, IFN-γ expression decreased significantly with the tumor stage progression. Based on a comprehensive analysis of the absolute counts, phenotypes, and functional markers of NK cells, we found an altered subset distribution and impaired function of circulating NK cells in patients with CRC.

## Introduction

Colorectal cancer (CRC) is a significant global health burden owing to its high incidence and mortality rate^1^. The continuous development of treatment technologies has enabled patients with early-stage CRC to achieve long-term survival through surgical tumor resection. However, those with advanced-stage CRC still have no chance of recovery^2,3^. Therefore, early preoperative diagnosis can provide vital information for treatment-related decision-making. Immune cell subsets are currently an exciting target for CRC immunotherapy and early clinical diagnostic biomarker research because immune system regulation influences disease progression in patients with CRC^4,5^.

Natural killer (NK) cells play an essential role in the antitumor immunity of immune cells because they quickly recognize and kill tumor cells without prior sensitization^6^. In peripheral blood, NK cells can be subdivided into three subsets based on their CD56 and CD16 expression levels: CD56^bright^, CD56^dim^CD16^+^, and CD56^dim^CD16^-^ NK cells. CD56^bright^ NK accounts for 5–10% of the total NK cells and is believed to be precursor cells of the CD56^dim^ subset^7,8^, which mainly participates in antitumor immune responses by secreting high levels of cytokines^9,10^. CD56^dim^CD16^+^ NK cells primarily induce cytotoxic activity and mediate target cell killing. They form the main subset of circulating NK cells, accounting for over 90%^11,12^. CD56^dim^CD16^-^ NK cells participate in immune regulation by releasing cytokines; however, they may inhibit the binding of NK cells to target cells^13,14^. In addition to changes in the distribution of effector NK cell subsets, impaired function of NK cells is closely associated with tumor progression. NK cells with impaired function are characterized by suppressed cytotoxic effects on tumor cells and reduced release of pro-inflammatory cytokines^15,16^.

Previous studies have explored the correlation between NK cells and the progression and metastasis of different types of malignant tumors^17,18^. Exploratory strategies for cancer immunotherapy using NK cells have been proposed^19,20^. However, research on the correlation between NK cells and CRC is limited. Therefore, this study aimed to comprehensively analyze the absolute counts, phenotypes, and functions of circulating NK cells in patients with CRC using multiparametric flow cytometry.

## Results

### The frequency and absolute count of NK cells were altered in the CRC group

In total, 107 patients in the CRC group (44 in the early stage and 63 in the advanced stage) and 182 in the control group were included in this study. Basic information on the study population is provided in Supplementary Table 1.

Compared to the control group, the frequency of NK cells in the CRC group decreased, and the distribution of NK cell subsets changed significantly, including the decreased frequency of CD56^dim^CD16^+^ NK cells, whereas the frequency of CD56^bright^ NK and CD56^dim^CD16^-^ NK cells increased (Table 1). Since the absolute counts of NK cells decreased significantly in the CRC group, CD56^dim^CD16^+^ and CD56^bright^ NK cells also decreased significantly. However, no significant difference in the absolute counts of CD56^dim^CD16^-^ NK cells was observed.

**Table 1:** Comparison of peripheral blood NK cell frequency and absolute counts between control and CRC groups.

	Control group N = 182	CRC group N = 107	*P* value
NK cell, % of lymphocytes	22.7 [15.1; 29.9]	18.7 [13.9; 27.0]	0.026*
CD56^bright^ NK, % of NK cell	2.11 [1.42; 3.39]	2.81 [1.69; 4.02]	0.010*
CD56^dim^CD16^+^ NK, % of NK cell	96.1 [94.0; 97.2]	94.2 [91.0; 96.0]	< 0.001*
CD56^dim^CD16^-^ NK, % of NK cell	1.50 [0.93; 2.31]	2.62 [1.79; 4.34]	< 0.001*
NK cell, 10^6^/L	397 [280; 572]	243 [158; 424]	< 0.001*
CD56^bright^ NK, 10^6^/L	8.94 [5.60; 12.7]	6.95 [4.29; 10.3]	0.001*
CD56^dim^CD16^+^ NK, 10^6^/L	376 [266; 559]	222 [141; 409]	< 0.001*
CD56^dim^CD16^-^ NK, 10^6^/L	5.73 [3.91; 8.89]	6.07 [3.57; 9.63]	0.669*

NK, Natural killer; CRC, colorectal cancer.

*: Wilcoxon rank-sum test was used for comparison between groups.

**Figure 1:**
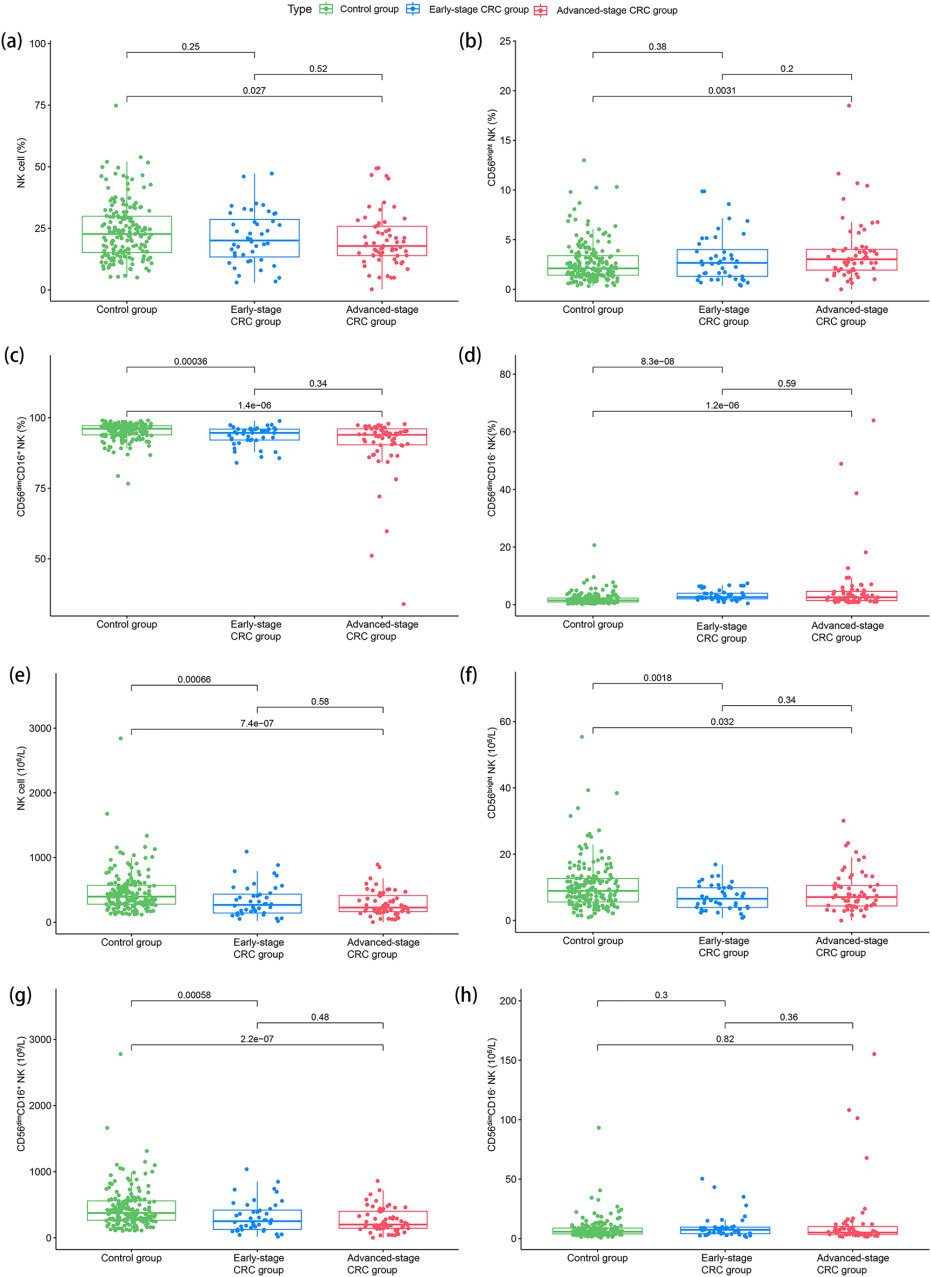
Distribution of peripheral blood NK cell subsets in the healthy control, early-stage CRC, and advanced-stage CRC groups. (**a**) Frequency of NK cells. (**b**) Frequency of CD56^bright^ NK cells. (**c**) Frequency of CD56^dim^CD16^+^ NK cells. (**d**) Frequency of CD56^dim^CD16^-^ NK cells. (**e**) Absolute NK cell counts. (**f**) Absolute counts of CD56^bright^ NK cells. (**g**) Absolute counts of CD56^dim^CD16^+^ NK cells. (**h**) Absolute counts of CD56^dim^CD16^-^ NK cells. Wilcoxon rank-sum test was performed for comparison among groups.

Figure 1 shows the relationship between the frequency and absolute counts of NK cells and the CRC stage. The statistical difference in cell frequencies between early and advanced-stage CRC was not noticeable, but the frequencies of NK and CD56^dim^CD16^+^ NK cells showed a downward trend in the control group, early-stage CRC, and advanced-stage CRC. In contrast, the frequencies of CD56^bright^ NK and CD56^dim^CD16^-^ NK cells showed an upward trend. The absolute counts of NK cells, CD56^bright^ NK cells, and CD56^dim^CD16^+^ NK cells were significantly decreased in early and advanced-stage CRC than in the control group. In contrast, no statistical significance was noted between early and advanced-stage CRC. In addition, there was no significant difference in the absolute count of CD56^dim^CD16^-^ NK cells between the control, early-stage CRC, and advanced-stage CRC groups.

### Intracellular interferon-γ secretion decreased in NK cells of the CRC group and was correlated with tumor stage

A total of 91 patients in the CRC group (50 in the early stage and 41 in the advanced stage) and 81 in the control group were included in this study. Basic information about the study population is provided in Supplementary Table 1.

**Figure 2:**
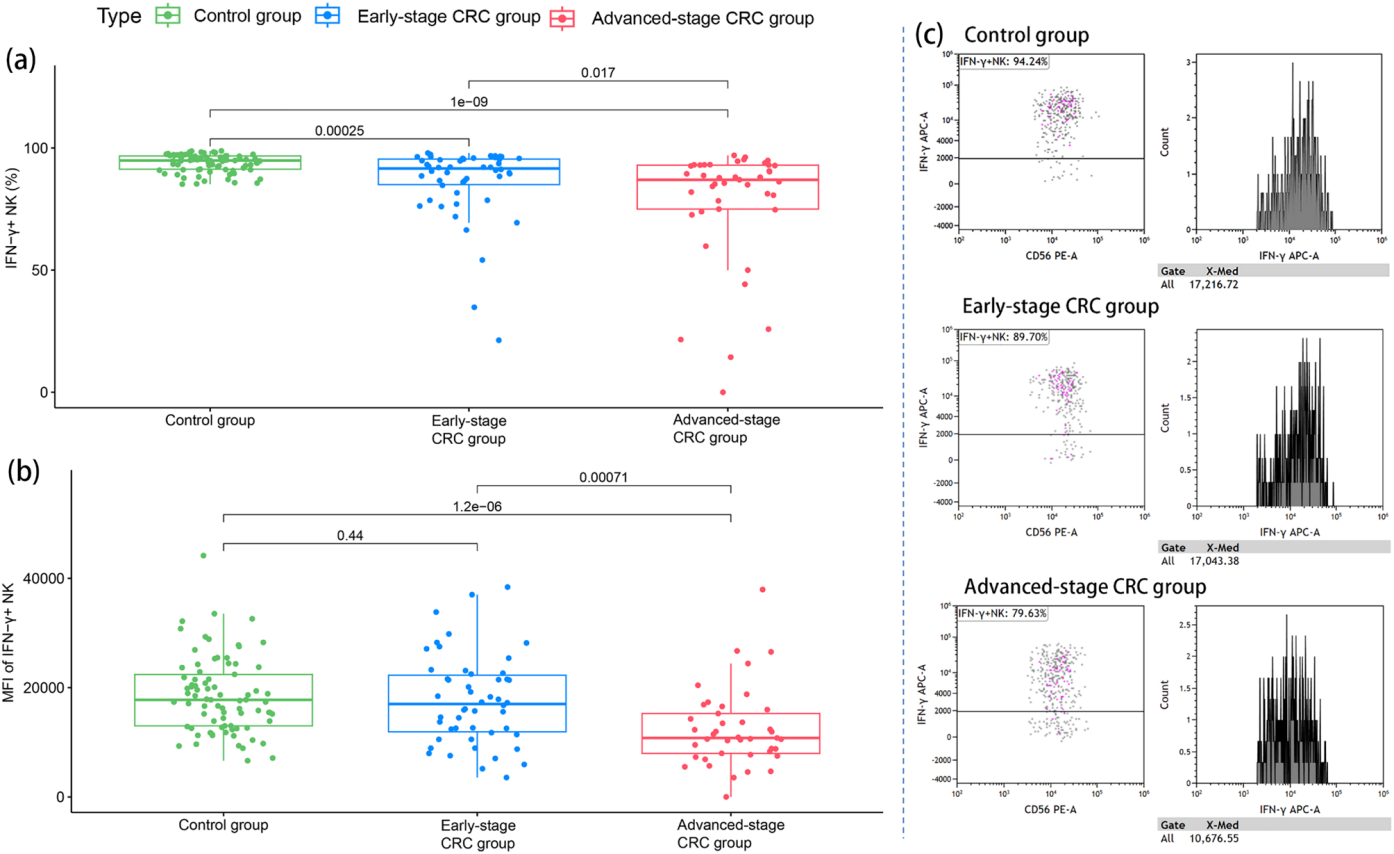
Intracellular IFN-γ secretion by NK cells in the healthy control, early-stage CRC, and advanced-stage CRC groups. (**a**) The percentage of IFN-γ^+^ NK cells in the healthy control, early-stage CRC, and advanced-stage CRC groups. (**b**) The MFI of IFN-γ^+^ NK cells in the healthy control, early-stage CRC, and advanced-stage CRC groups. (**c**) Intracellular IFN-γ secretion levels by NK cells in one representative healthy control, one representative patient with early-stage CRC, and another with advanced-stage CRC. Wilcoxon rank-sum test was performed for comparison among groups.

Compared to the control group, the percentage of intracellular interferon (IFN)-γ^+^ NK cells was significantly reduced in the CRC group (89.5 [78.6–94.6] vs. 94.9 [91.3–96.8], *P* < 0.001) and was accompanied by a decrease in the median fluorescence intensity (MFI) of IFN-γ^+^ NK cells (13,666.5 [8928.8–20,436.8] vs. 17,767.3 [13001.0–22,423.1], *P* = 0.001). Moreover, the percentage of intracellular IFN-γ^+^ NK cells in the CRC group correlated with the disease stage (Fig. 2a). However, the reduced MFI of IFN-γ^+^ NK cells was not significant in patients with early-stage CRC and was observed mainly in patients with advanced-stage CRC (Fig. 2b).

Figure 2c showed the intracellular IFN-γ secretion levels by NK cells in one representative healthy control, one representative patient with early-stage CRC, and another with advanced-stage CRC.

### The positive percentage of granzyme B and perforin on the surface of NK cells were significantly decreased in the CRC group

A total of 66 patients in the CRC group (38 in the early stage and 28 in the advanced stage) and 58 in the control group were included in this study. Basic information about the study population is provided in Supplementary Table 1.

**Figure 3:**
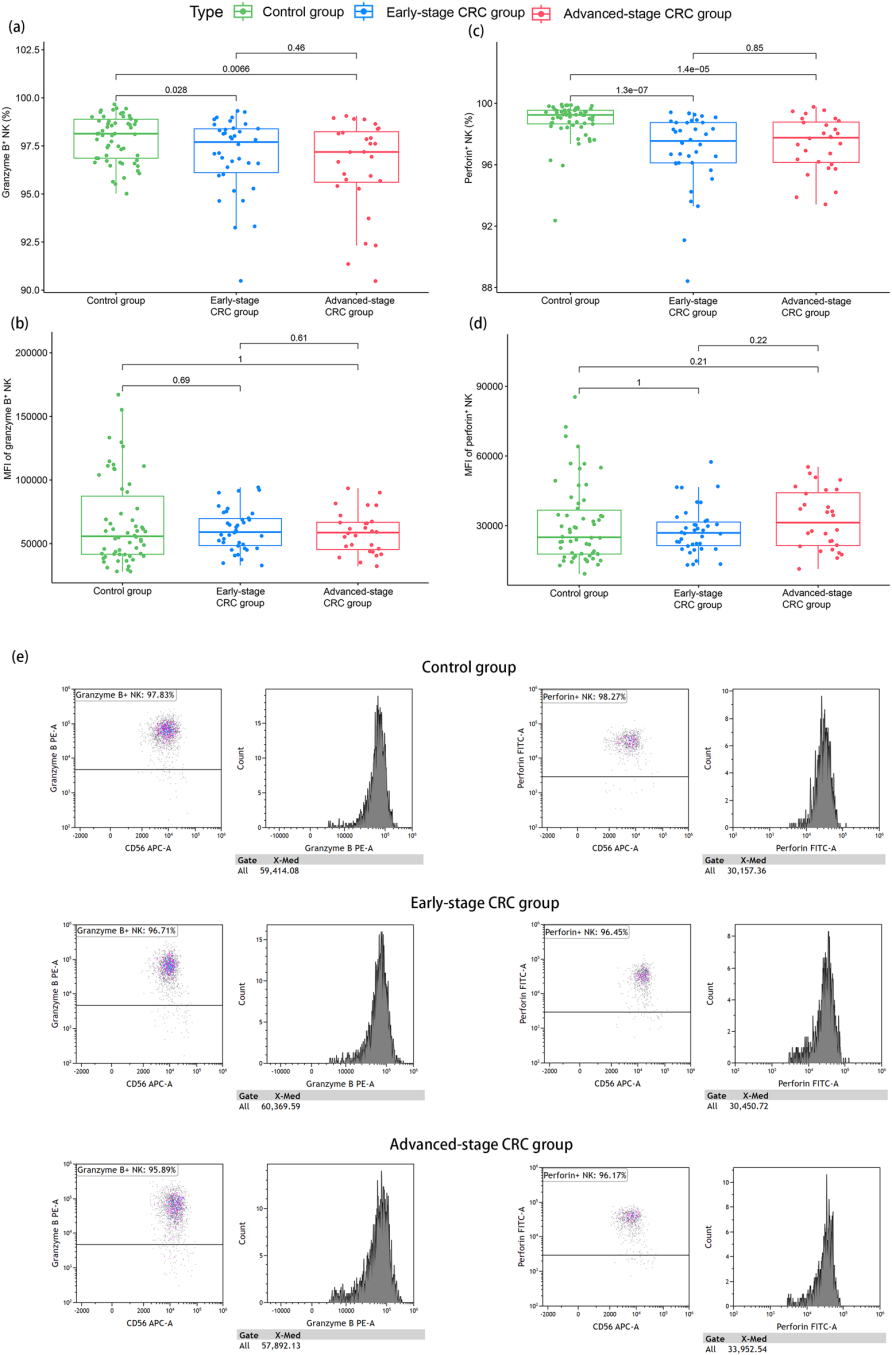
Expression levels of granzyme B and perforin on the surface of NK cells in the healthy control, early-stage CRC, and advanced-stage CRC groups. (**a**) The percentage of granzyme B^+^ NK cells in healthy control, early-stage CRC, and advanced-stage CRC groups. (**b**) The MFI of granzyme B + NK cells in healthy control, early-stage CRC, and advanced-stage CRC groups. (**c**) The percentage of perforin^+^ NK cells in healthy control, early-stage CRC, and advanced-stage CRC groups. (**d**) The MFI of perforin^+^ NK cells in healthy control, early-stage CRC, and advanced-stage CRC groups. (**e**) Expression levels of granzyme B and perforin on the surface of NK cells in one representative healthy control, one representative patient with early-stage CRC, and another with advanced-stage CRC. Wilcoxon rank-sum test was performed for comparison among groups.

Compared to the control group, the positive percentage of granzyme B on the surface of NK cells was significantly decreased (97.4 [95.8–98.4] vs. 98.1 [96.9–98.9], *P* = 0.003). In addition, as shown in Fig. 3a, the difference in granzyme B percentage on the surface of NK cells between the early and advanced-stage CRC groups was insignificant. Furthermore, no significant difference was observed in the MFI of granzyme B^+^ NK cells among the control, early- and advanced-stage CRC groups (Fig. 3b).

Compared to the control group, the positive percentage of the surface perforin of NK cells was significantly reduced (97.7 [96.1–98.8] vs. 99.2 [98.7–99.6], *P* < 0.001). In addition, as shown in Fig. 3c, the surface perforin percentage of NK cells was not significantly different between early and advanced-stage CRC. In addition, there was no significant difference in the MFI of perforin^+^ NK cells among the control, early-, and advanced-stage CRC groups (Fig. 3d).

Figure 3e showed the expression levels of granzyme B and perforin on the surface of NK cells in one representative healthy control, one representative patient with early-stage CRC, and another with advanced-stage CRC.

## Discussion

Recent studies have shown that NK cell populations are highly heterogeneous^6,21^. This study divided NK cells into three subpopulations using CD56 and CD16 markers. We found that the phenotype, absolute counts, and function of NK cells were significantly altered in the peripheral circulation of patients with CRC, mainly manifesting as a decrease in the absolute counts of NK cells, a change in the frequency of NK cell subsets, a decrease in the percentage and MFI of intracellular IFN-γ of NK cells, and a significant decrease in the positive percentage of granzyme B and perforin on the cell surface.

CD56^bright^ NK cells are thought to be likely precursor cells for subsets of CD56^dim^ NK cells, which are involved in immune regulation by producing high levels of cytokines that can inhibit the proliferation of CD4^+^T cells^22^ and play essential roles in implantation, angiogenesis, and pregnancy maintenance^23^. However, the antitumor response of CD56^bright^ NK cells is considered ineffective^24,25^. Other malignant tumors have reported an increased proportion of peripheral circulating CD56^bright^ NK cells^26,27^. NK cells initiate antibody-dependent NK cell activation through Fc gamma receptor IIIA/CD16 expression^28^; thus, the CD56^dim^CD16^+^ NK cell subset represents effector cells with tumor-killing functions. In contrast, the absence of CD16 renders the CD56^dim^CD16^-^ NK cell subset much less capable of executing antibody-dependent cell-mediated cytotoxicity than the CD56^dim^CD16^+^ NK cell subset^15,29^. Previous studies have shown that the proportion or absolute value of CD56^dim^CD16^-^ NK cell increases in patients with CRC^30,31^. The decreased frequency of CD56^dim^CD16^+^ NK cells with antitumor effects and the increased frequency of CD56^bright^ and CD56^dim^CD16^-^ NK cells with poor or ineffective antitumor effects in patients with CRC indicate a decrease in the antitumor capacity of NK cells in patients with CRC. Moreover, a decrease in the total number of NK cells further exacerbates the downregulation of the ability of NK cells to induce antibody-dependent, cell-mediated cytotoxicity. In addition, the lower absolute count and maturity of circulating NK cells in patients with CRC may be associated with the redistribution of antitumor cells within the body, where mature and cytotoxic NK cells potentially migrate to tissues to combat the tumor.

The expression of cytotoxic effector molecules, including granzyme B, perforin, and the cytokine IFN-γ can reflect NK cell function. Granzyme B and perforin are potent anticancer mediators that can control tumor proliferation and spreading. The interaction of granzyme B with perforin produces potent cytolytic functions, leading to tumor cell lysis^32,33^. IFN-γ plays a role in inhibiting cell proliferation and promoting apoptosis of cancer cells^34^. Hodge et al.^35^ noted reduced NK cell granzyme B, perforin, and IFN-γ expression in lung cancer. Similar to previous tumor findings, we also found a reduced percentage and MFI of NK cells IFN-γ, and a reduced positive percentage of granzyme B and perforin in circulating NK cells in patients with CRC, which may also favor cancer cell survival.

This study conducted a three-part experiment to demonstrate changes in the distribution and impaired function of circulating NK cells in patients with CRC. To the best of our knowledge, this is the first study to quantify the circulating antitumor immunity of NK cells in patients with CRC by combining the frequency of NK cell subset distribution, absolute counts, and markers of NK cell functions. In a previous study, Krijgsman et al.^36^ investigated the phenotypes of circulating immune cell subsets in CRC. They found that the differences in peripheral blood immune cell profiles were mainly related to the presence of colorectal tumors rather than tumor stage. We further found that the frequency and absolute counts of NK cell subset distribution and the percentage of granzyme B and perforin were not related to tumor stage, but the percentage and MFI of IFN-γ were significantly decreased with the progression of tumor stage. Therefore, analysis of the absolute count or phenotype of NK cells alone does not represent the immune status of NK cells. A comprehensive analysis of the absolute counts, phenotypes, and functional markers of NK cells would be conducive to a better understanding of the immune status of circulating NK cells in patients with CRC.

This study has some limitations. The effect of FCGR3A F158V genotypic polymorphism on the affinity between clone 3G8 and CD16 was not further investigated based on the genotype, although the antibodies were selected based on the optimal titer after titration experiments, and the genotypes of all study subjects were randomized. In addition, this is a single-center study that only analyzed circulating NK cells. Further efforts will be directed at a multicenter study incorporating the tumor microenvironment.

In conclusion, based on a comprehensive analysis of the absolute counts, phenotypes, and functional markers of NK cells, this study revealed an altered subset distribution and impaired function of circulating NK cells in patients with CRC.

## Materials and methods

### Study population

Patients in the CRC group were prospectively recruited based on their colonoscopy results. The inclusion criteria used are masses detected by colonoscopy that required a pathological diagnosis, ≥ 18 years, no other malignancy or autoimmune disease, and no relevant surgical diagnosis or treatment. Peripheral blood samples collected within two weeks before surgery or other treatments were temporarily processed. Patients finally included in this study had primary CRC confirmed by histopathology and no known bacterial or viral infection. According to the tumor node metastasis (TNM) classification criteria, the final study group was divided into early-stage (TNM stages I and II) and advanced-stage (TNM stages III and IV). The control group comprised healthy volunteers who were matched for sex and age and were examined at the Physical Examination Center of the Affiliated Dongyang Hospital of Wenzhou Medical University during the same period.

The Ethics Committee of the Affiliated Dongyang Hospital of Wenzhou Medical University approved this study (Approval number: 2020-YX-100). Written informed consent was obtained from all the prospectively enrolled patients and healthy volunteers. All methods of this study were performed in accordance with the relevant guidelines and regulations.

### Detection of phenotype and absolute counts in NK cell subsets

The population included in the NK cell phenotype and absolute count studies was recruited from the Affiliated Dongyang Hospital of Wenzhou Medical University between July 2021 and March 2023. The following sample processing steps were briefly performed before flow cytometry detection: fresh whole blood (100 µL) was mixed thoroughly with the appropriate antibodies (Supplementary Table 2) and incubated for 15 min. Next, 2 mL of OptiLyse B (Beckman Coulter) was added and incubated for another 15 min to lyse the red blood cells completely. After centrifuging the tube at 400 × g for 5 min, the supernatant was removed, and 250 µL of phosphate buffer solution was added for instrument detection. A ten-color flow cytometer (Navios; Beckman Coulter) was used to detect the NK cell phenotypes. The gating strategy is illustrated in Supplementary Fig. 1a.

Total lymphocyte count was determined using a hematology analyzer (XN-9000; SYSMEX, Japan). The absolute count of each subset was calculated according to the proportion of NK cells and their subsets in the lymphocytes.

### Detection of IFN-γ secretion in NK cells

The study population for intracellular IFN-γ secretion in NK cells was recruited at the Affiliated Dongyang Hospital of Wenzhou Medical University from September 2022 to December 2023. The method for measuring intracellular IFN-γ secretion in NK cells was based on the study reported by Tang et al.^37^. The flow of the sample processing is shown in Supplementary Fig. 2. Specific information regarding the antibodies used is provided in Supplementary Table 1. Finally, intracellular IFN-γ secretion by the NK cells was detected using a 13-color flow cytometer (DxFlex; Beckman Coulter). The gating strategy is illustrated in Supplementary Fig. 1b.

### Detection of surface granzyme B and perforin expression levels in NK cells

The study population for investigating NK cell surface granzyme B and perforin expression levels was recruited from February 2023 to December 2023 at the Affiliated Dongyang Hospital of Wenzhou Medical University. The sample processing steps before flow cytometry were similar to the above-mentioned steps for the phenotypic detection of NK cell subsets. Information on the monoclonal antibodies used in this study is presented in Supplementary Table 2. Finally, the expression of surface granzyme B and perforin in NK cells was detected using, a 13-color flow cytometer (DxFlex; Beckman Coulter). The gating strategy is illustrated in Supplementary Fig. 1c.

### Statistical analysis

Kaluza software (version 2.1, Beckman Colter) was used to analyze flow cytometry data. SPSS (version 23.0; IBM, Chicago, IL, USA) and R software version 4.3.2 (R Foundation for Statistical Computing) were used for statistical analysis and data visualization. Continuous data are expressed as the mean ± standard deviation or median (interquartile range).

The differences between the groups was analyzed using the two-tailed unpaired Student’s t-test or Wilcoxon rank-sum test. Statistical significance was set at *P* < 0.05.

### Ethics approval statement

The Ethics Committee of the Affiliated Dongyang Hospital of Wenzhou Medical University approved this study (Approval number: 2020-YX-100) .

### Consent to participate

All prospectively enrolled patients and healthy volunteers provided their informed consent.

### Supplementary Information

Supplementary Information.

## Data Availability

The data underlying this article are available in the article and its online supplementary material.
