# Supplementary material for: Changes in subset distribution and impaired function of circulating natural killer cells in patients with colorectal cancer
Source: Sci Rep. 2024 May 28;14:12188. doi: 10.1038/s41598-024-63103-x (PMC11133342; doi:10.1038/s41598-024-63103-x)
Supplement: Supplementary file 1 — Supplementary Information. [file 41598_2024_63103_MOESM1_ESM.pdf]

Changes in subset distribution and impaired function of circulating natural killer cells in patients with colorectal cancer

Shujin Zu[1#], Yan Lu[2#], Rui Xing[3], Xiang Chen[4]， Longyi Zhang[2*]

[1]Department of Reproductive Center, Affiliated Dongyang Hospital of Wenzhou Medical University, 60 West Wuning Road, Dongyang 322100, Zhejiang, China; [2]Clinical Laboratory, Affiliated Dongyang Hospital of Wenzhou Medical University, 60 West Wuning Road, Dongyang 322100, Zhejiang, China; [3]The Department of Hematology, Affiliated Dongyang Hospital of Wenzhou Medical University, Zhejiang, China; [4]Department of Biomedical Sciences Laboratory, Affiliated DongYang Hospital of Wenzhou Medical University, Zhejiang, China

[#]These authors contributed equally to this work

***Corresponding author:** Longyi Zhang, Clinical Laboratory, DongYang People's Hospital, 60 West Wuning Road, Dongyang 322100, Zhejiang, China

E-mail: happy_zhang1y@163.com (LZ)

Supplementary Table 1 Basic characteristics of the control and study groups.

	A study		B study		C study	
	Control group (N = 182)	Study group (N = 107)	Control group (N = 81)	Study group (N = 91)	Control group (N = 58)	Study group (N = 66)
Age (years)	64.5 ± 14.9	61.8 ± 11.4	67.3 ± 13.5	66.9 ± 9.8	67.7 ± 12.6	67.0 ± 9.3
Sex						
Male	91 (50.0%)	57 (53.3%)	49 (60.5%)	54 (59.3%)	35 (60.3%)	38 (57.6%)
Female	91 (50.0%)	50 (46.7%)	32 (39.5%)	37 (40.7%)	23 (39.7%)	28 (42.4%)
TNM stage						
Stage I		11 (10.3%)		20 (22.0%)		15 (22.7%)
Stage II		33 (30.8%)		30 (33.0%)		23 (34.8%)
Stage III		46 (43.0%)		29 (31.9%)		19 (28.8%)
Stage IV		17 (15.9%)		12 (13.2%)		9 (13.6%)
Tumor site						
Colon		38 (35.5%)		36 (39.6%)		26 (39.4%)
Rectum		69 (64.5%)		55 (60.4%)		40 (60.6%)

A study: Study of NK cell subset phenotypes and absolute counts;
B study: Study of intracellular interferon (IFN)-γ secretion by NK cells;
C study: Study on the expression levels of granzyme B and perforin on the surface of NK cells.
TNM: tumor-node-metastasis.

Supplementary Table 2 Three staining panels for monoclonal fluorescent antibody labelling of circulating NK cell phenotype and function.

Fluorochrome	A panel for NK cell subsets phenotypic detection			A panel for detection of intracellular IFN-γ secretion of NK cells			A panel for detection of surface granzyme B and perforin expression levels in NK cells		
	Marker	Clone	Source	Marker	Clone	Source	Marker	Clone	Source
FITC	CD20/ CD3/ CD19	clone B9E9/ clone UCHT1 /clone J4.119	Beckman Coulter				Perforin	clone B-D48	Biolegend
PE				CD56	clone N901	Beckman Coulter	Granzyme B	clone QA16A02	Biolegend
PC5.5				CD3	clone UCHT1	Beckman Coulter	CD8	clone B9.11	Beckman Coulter
PE-Cy7							CD3	clone UCHT1	Beckman Coulter
APC				IFN-γ	clone 4S.B3	Biolegend	CD56	clone N901	Beckman Coulter
AA750							CD4	clone 13B8.2	Beckman Coulter
AA700	CD56	clone N901	Beckman Coulter						
PB	CD16	clone 3G8	Beckman Coulter						
KRO	CD45	clone J.33	Beckman Coulter	CD45	clone J.33	Beckman Coulter	CD45	clone J.33	Beckman Coulter

FITC, Fluorescein Isothiocyanate; PE, Phycoerythrin; PC5.5, Phycoerythrin-Cyanin 5.5; PE-Cy7, Phycoerythrin-Cyanin 7; APC, Allophycocyanin; AA750, APC- Alexa Fluor 750; AA700, APC-Alexa Fluor 700; PB, Pacific Blue; KRO, Krome Orange

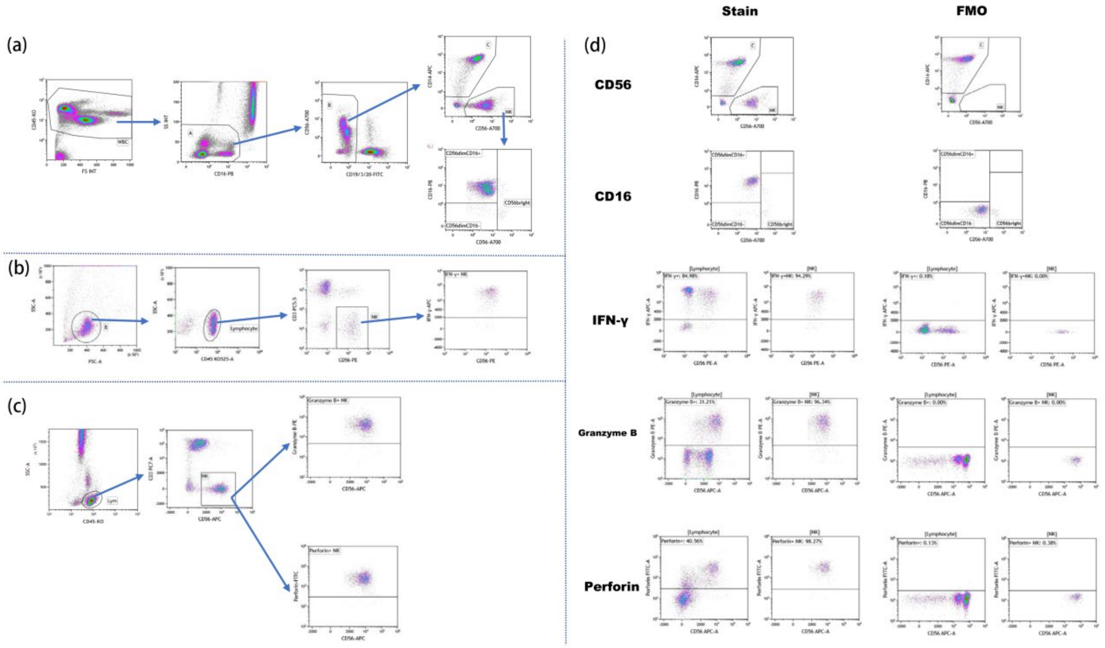

Supplementary Figure 1 Gating strategy for NK cell phenotype and function detection. (a) Gating strategy for NK cell phenotype detection; (b) Gating strategy for NK cell intracellular interferon (IFN)-γ secretion detection; (c) Gating strategy for NK cell surface granzyme B and perforin expression level detection; (d) FMO controls for CD56, CD16, IFN-γ, granzyme B and perforin were evaluated. FMO: fluorescence minus one.

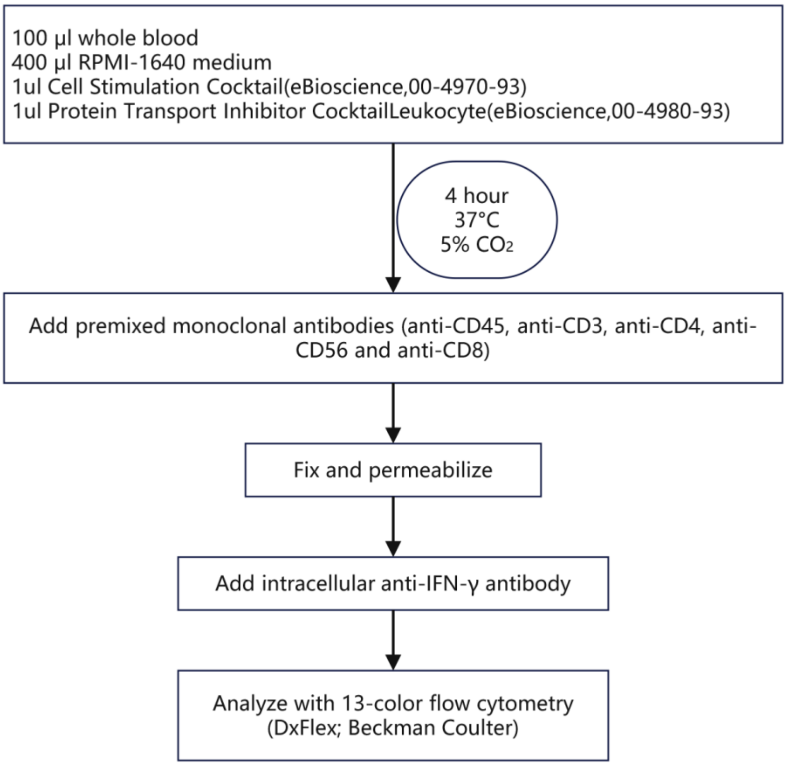

Supplementary Figure 2 Sample processing procedure for intracellular IFN-γ secretion in NK cells.
